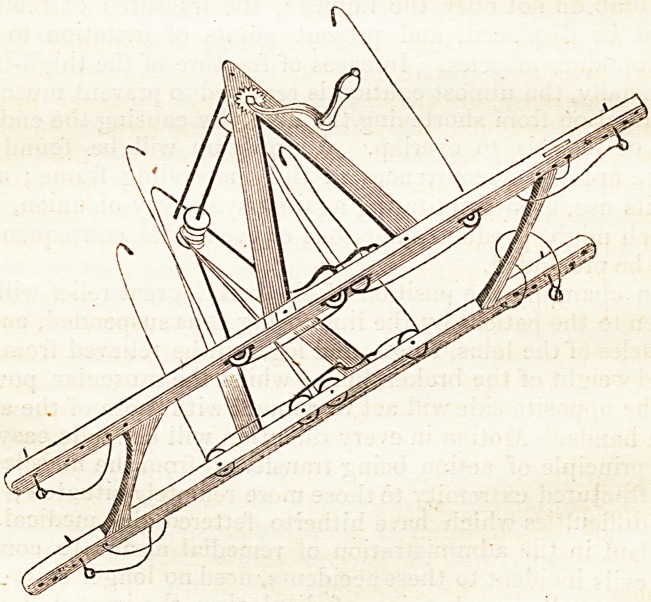# On the Treatment of Fractures of the Bones of the Lower Extremity; with a Description of an Improved Apparatus for Suspending the Limb

**Published:** 1827-03

**Authors:** William Chandler

**Affiliations:** Surgeon of the Kent and Canterbury Hospital.


*On the Treatment of Fractures of the Bones of the Lower Extremity; with a Description of an improved Apparatus for suspending the Limb.* By William Chandler, Esq. Surgeon of the Kent and Canterbury Hospital.

To enter into a detail of the various methods which have been proposed by the most eminent men of the faculty, in this and in other countries, for keeping in apposition the fractured extremities of bones of the lower limbs, would be foreign to the purport of this communication; and equally so to combat the diversity of opinion existing on the best position for placing the limb.

On one point, however, all parties appear to concur,—viz. the absolute necessity of guarding against disturbance of the fractured bone, during the process of union. Amidst the infinity of plans which have been suggested to attain so desirable an event, the ingenious contrivance of a foreign surgeon has not sufficiently arrested the attention of the medical world. His proposal—that of suspending the limb during the progression of union, in cases of fracture of the lower extremities,—has for its avowed objects to expedite the cure, to afford the utmost attainable comfort to the patient, and to avert the calamities often ensuing from accidents of this nature.

I am indebted to my friend, Mr. George Young, for his communication of the favourable termination of such accidents, when treated according to a system advised by an eminent surgeon of one of the Swiss hospitals, whose name has escaped my recollection, but with whom success has been almost unvaried, even in most frightful cases.

If mechanical contrivance can be so adapted as to be the mean of mitigating the sufferings of those who are deprived by accident of the power of motion, and by its proper application of diminishing, if not totally precluding, the frequent appalling consecutive evils, it must be an object of the first consideration: nor should the mind be prejudiced against an invention, originating from whence it may. The object this Swiss surgeon had in view, when recommending the limb to be suspended in cases of fracture of the lower extremity, was to afford greater facility in the movement of the person whilst submitting to necessary operations, with the least possible disturbance to the limb;—to supersede extra assistance;—to allow of variation in the position of the body, without calling into action the muscles of the fractured extremity, or giving the slightest interruption to the fractured bones when once in a state of apposition; and consequently to render the patient exempt from the train of miseries attending nervous excitement, when the ends of the fractured bone cannot be preserved in a perfect state of adjustment by the ordinary mode of placing the limb, and to afford more ample means of attending to the comfort of the patient during the time of dressing wounds connected with, or in the vicinity of, the fracture, and in the change of linen, &c. &c.

The frame for suspending limbs, which I have used for the last four years, would appear, in many cases of fracture of the lower extremity, to possess decided advantages over the moveable bedstead of Mr. Earle; the weight, size, and expence of which renders its use inapplicable in many situations, and with the generality of persons in whom such accidents occur. On the contrary, the simplicity of the construction, portability, and cheapness of the suspending frame, of which a drawing is annexed, will allow of its application to any bed, and in any situation.

In all cases of fracture of the lower limb, in proportion to the extent of injury done to the bone and to the surrounding soft parts,—more especially in those of the compound, the oblique, and complicated kind,—will suspension be found of importance. Its chief value will be found in counteracting, as before stated, the causes which produce local disturbance, and subsequent constitutional irritation; as it ensures the perfect quietude of the limb, whilst its natural and easiest position can be guaranteed. In order to accomplish the important objects, splints varying in form and complexity have been introduced, and, by their judicious management, success has frequently been obtained. The least complicated form of these auxiliaries to the union of the bone will still be instrumental in the recovery of the patient; but I believe that splints of any description, however ingeniously invented and accurately applied, in many cases of compound fractures, or fractures giving rise to spasmodic action or great excitement of the system, will be held in the lowest estimation, unless their application be aided by some machinery or apparatus, which will secure the fractured bones from being displaced by more than ordinary action of the muscles. Suspension will accomplish this desideratum,—and the limb, being thus freed from the incumbrance and impediments offered by the bedding and clothing surrounding it, if placed in the usual mode, will be allowed such latitude of action as to enable the patient to shift his position without hazard to the fracture, or fear of exciting irritation; since no movement of the body can take place without the fractured limb being in corresponding action with it.

When the fractured limb, according to the usual method of arrangement, is made a fixture to the bed, one of the most perplexing consequences of the involuntary movements of the patient whilst lying, is that it slides from the pillow to the foot of the bed. Thus the upper portion of the bone forces the parts beneath the fracture onwards. If the lower part of the limb do not obey the impulse, the fractured extremities must be displaced, and present points of irritation to the surrounding muscles. In cases of fracture of the thigh-bone especially, the utmost caution is required to prevent muscular contraction from shortening the limb, by causing the ends of the os femoris to overlap. A provision will be found for these untoward occurrences in the suspending frame; and, by its use, events militating against symmetry of union, and which might produce dangerous or even fatal consequences, will be precluded.

In changing the position of the body, great relief will be given to the patient by the limb being thus suspended, as the muscles of the loins, thigh, and leg, will be relieved from the dead weight of the broken limb; whilst the muscular powers of the opposite side will act in concert with those of the arms and hands. Motion in every direction will be made easy by the principle of action being transferred from the muscles of the fractured extremity to those more remotely situated; and the difficulties which have hitherto fettered the medical attendant in the administration of remedial means to combat the evils incident to these accidents, need no longer exist; for, I repeat, all apprehension of displacing the impacted bone may be banished when the limb is once suspended.

The height to which it may be necessary to support the limb by the frame, must vary according to circumstances:

generally about half an inch just clear from the bedding will be sufficient to allow the patient, in volition, to move higher or lower in the bed, as best suits his comforts.

In the removal of under-linen, or in the use of the night-pan, the limb may be raised to that height which is most convenient. Suspending the limb will not interfere with the ordinary mode of disposing the limb, nor with the apparatus considered most efficient.

In cases of disease of the hip, knee, and ankle-joints, where perfect rest is often one of the most salutary measures, the application of the suspending frame will not be less advantageous than in those cases to which I have now more particularly referred.

A frame for the convenience of patients must, in the construction, in some measure depend on the form of the top of the bed. That which has been most convenient in my public and private practice was planned to suit the iron bedsteads of the Kent and Canterbury Hospital; but the frame can be equally well supported by four uprights of common deal wood, with two rails or cross bars on the top, on which it may rest.

The subjoined plan will convey to any mechanic a representation of the frame I have in use; and the contrivance for suspending the lower limbs, in use at the Kent and Canterbury

Hospital, consists of a frame made of beech, three feet six inches in length, with four brass pulleys in each side: at the extremity pin-holes are bored, for the insertion of iron or brass pins, to secure the frame on the bed-top. Within eight inches of the frame, two rails, of twenty-three inches, are placed to connect it. Two side frames are screwed into the upper frame, of twenty-one inches in length, and brought to an angle at the bottom, through which is passed an iron rod, having two wooden pulleys or rollers, into which four green lines are inserted, with hooks at the extremity, by which the fracture-box is to be attached and suspended. At one end of the iron rod is a handle of wood, with a turning notch, and iron stop in the side frame: by means of this rod, the fracture-box may be raised or lowered at pleasure. At the end of the angular frame an iron rod is bolted, for making the lower part of the apparatus secure.

*Canterbury; September 9th,* 1826.

## Figures and Tables

**Figure f1:**